# Psychiatric disorders in patients after hospitalization for COVID-19: Frequency, coping behaviours and associated factors

**DOI:** 10.1192/j.eurpsy.2024.1054

**Published:** 2024-08-27

**Authors:** N. Sghaier, H. Ben Garouia, H. Khiari, W. Belaguide, J. Mannai, H. Bellali

**Affiliations:** ^1^Psychiatry Departement, Ibn Jazzar Hospital, Kairouan; ^2^Preventive and Community Departement, Habib Thameur Hospital, Tunis, Tunisia

## Abstract

**Introduction:**

The COVID-19 pandemic caused an unprecedented major health crisis. Current data suggest that psychiatric sequelae may persist for a long time in survivors after infection.

**Objectives:**

The objectives of our study were to determine the frequency of anxiety, depression, sleep disorders, and posttraumatic stress disorder in patients after hospitalization for COVID-19 infection, and to investigate factors associated with their occurrence as well as to identify coping behaviors in these patients.

**Methods:**

This was a descriptive cross-sectional study conducted at Ibn Jazzar Kairouan Hospital between September and December 2021 among patients who consulted three months after their hospitalizations for COVID-19 infection. The assessment of the different psychiatric disorders was performed using the validated Arabic versions of the Hospital Anxiety and Depression Scale, Post-traumatic Stress Disorder Checklist for DSM-5 and the Pittsburgh Sleep Quality Index. Coping behaviors were studied using the Brief-COPE scale.

**Results:**

Our work included 104 patients. The median age was 55.5 years [IQR:47-64]. The gender ratio M/F was 1.12. Anxiety and depressive symptoms were found in 26.9% and 25% of cases, respectively. The frequency of post-traumatic stress disorder was 22.1% and that of sleep disorders was 41.3%. Problem-solving strategies were the most widely adopted, followed by emotion-focused strategies. Younger age, female gender, persistence of a physical symptom, impairment of daily activity, and stigma were factors independently associated with psychological distress. No association was found between the intensive care unit stay and psychiatric disorders. Problem-focused and emotion-focused coping were negatively correlated with the different psychological outcomes studied.

**Conclusions:**

Psychological distress in COVID-19 survivors persists beyond the acute phase and results from an intricacy of several factors. This highlights the importance of regular psychiatric follow-up after hospitalization in order to identify and treat, as early as possible, psychiatric disorders.

**Disclosure of Interest:**

None Declared

